# A population of Pax7-expressing muscle progenitor cells show differential responses to muscle injury dependent on developmental stage and injury extent

**DOI:** 10.3389/fnagi.2015.00161

**Published:** 2015-08-25

**Authors:** Stefanie Knappe, Peter S. Zammit, Robert D. Knight

**Affiliations:** ^1^Department of Craniofacial Development and Stem Cell Biology, Dental Institute, King's College LondonLondon, UK; ^2^Randall Division of Cell and Molecular Biophysics, Faculty of Life Sciences and Medicine, King's College LondonLondon, UK

**Keywords:** regeneration, stem cells, myogenesis, zebrafish, satellite cells, skeletal muscle, wound healing

## Abstract

Skeletal muscle regeneration in vertebrates occurs by the activation of quiescent progenitor cells that express *pax7* to repair and replace damaged myofibers. We have developed a mechanical injury paradigm in zebrafish to determine whether developmental stage and injury size affect the regeneration dynamics of skeletal muscle. We found that both small focal injuries, and large injuries affecting the entire myotome, lead to expression of *myf5* and *myogenin*, which was prolonged in older larvae, indicating a slower process of regeneration. We characterized the endogenous behavior of a population of muscle-resident Pax7-expressing cells using a *pax7a*:eGFP transgenic line and found that GFP+ cell migration in the myotome dramatically declined between 5 and 7 days post-fertilization (dpf). Following a small single myotome injury, GFP+ cells responded by extending processes, before migrating to the injured myofibers. Furthermore, these cells responded more rapidly to injury in 4 dpf larvae compared to 7 dpf. Interestingly, we did not see GFP+ myofibers after repair of small injuries, indicating that *pax7a*-expressing cells did not contribute to myofiber formation in this injury context. On the contrary, numerous GFP+ myofibers could be observed after an extensive single myotome injury. Both injury models were accompanied by an increased number of proliferating GFP+ cells, which was more pronounced in larvae injured at 4 dpf than 7 dpf. This indicates intriguing developmental differences, at these early ages. Our data also suggests an interesting disparity in the role that *pax7a*-expressing muscle progenitor cells play during skeletal muscle regeneration, which may reflect the extent of muscle damage.

## Introduction

Skeletal muscle is a complex tissue consisting of multinuclear, contractile muscle fibers, yet it has a robust regenerative capacity through the action of muscle stem cells (muSCs). In mouse, the principle cells responsible for repairing or replacing damaged muscle are satellite cells (SCs). These were first described in frog and rat in 1961 (Mauro, 1961), and have since been described in most vertebrate species, including fish and other rodents (Moss and Leblond, 1970; Schultz et al., 1978; Koumans et al., 1990; Stoiber and Sänger, 1996). Generally, these tissue-resident stem cells are located beneath the basal lamina of muscle fibers and show a characteristically large, heterochromatic nucleus in their quiescent state. Genetically, quiescent muSCs are characterized by expression of the paired homeobox transcription factor Pax7 (Seale and Rudnicki, 2000). It has been shown that Pax7-deficient muSCs show a loss of heterochromatin in their nucleus, suggesting the transcription factor is essential for maintaining quiescence (Gunther et al., 2013).

Pax7-expressing muSCs originate in the dermomyotome during embryogenesis, which contributes to the formation of skeletal muscle tissue in the trunk (Kassar-Duchossoy et al., 2004; Gros et al., 2005; Relaix et al., 2005). Their activation in post-natal animals can be induced by many stimuli, most notably mechanical injury. Activated and proliferating muSCs are characterized by the expression of the myogenic regulatory factors (MRFs) *Myf5* and *MyoD* and later express *Myogenin* upon differentiation. A subset of these cells can undergo asymmetric cell division, giving rise to one progenitor and one differentiating daughter cell (Zammit et al., 2004; Shinin et al., 2006; Conboy et al., 2007). These cells are essential for maintenance and repair of skeletal muscle tissue and loss of Pax7 results in an impaired regenerative response after injury (Lepper et al., 2011; Sambasivan et al., 2011; Von Maltzahn et al., 2013). They regenerate injured muscle predominantly by either fusing to damaged muscle fibers, or to each other to form *de novo* muscle fibers. In mammals, newly formed myofibers generally have a smaller diameter and show myonuclei located centrally, as opposed to their usual location at the periphery of the myofiber.

Much of our understanding of how skeletal muscle regenerates comes from studies performed in the mouse. In fish, the presence of muSCs has been demonstrated in adult muscle tissue in a number of species including salmon, carp, and electric fish (Nag and Nursall, 1972; Akster, 1983; Weber et al., 2012). Extraction of muSCs from adult zebrafish also reveals that these cells show immunoreactivity for Pax7 and can form muscle fibers in culture (Alexander et al., 2011; Zhang and Anderson, 2014). Tissue regeneration in adult zebrafish has been described to occur within 28 days and involves the formation of regenerative fibers in conjunction with BrdU labeling, indicating proliferating progenitor cells (Rowlerson et al., 1997).

Investigations into the developmental origin of *pax7*+ cells in zebrafish larvae revealed that they originate from the dermomyotome, similarly to amniotes (Hollway et al., 2007; Marschallinger et al., 2009). These cells are mitotically inactive and express several typical muSC markers, such as *cxcr4* genes (Hollway et al., 2007) and Syndecan-4 (Froehlich et al., 2013). Further, muscle regeneration occurs through *de novo* formation of new fibers and not, as previously assumed, by de-differentiation in larval animals (Rodrigues et al., 2012). Further, muSCs have also been shown to respond to injury stimuli by migrating to, and proliferating at, the site of injury in zebrafish larvae (Seger et al., 2011; Otten et al., 2012).

The majority of studies examining muSC function have been performed in mouse using models, such as cardiotoxin or barium chloride, inducing fairly major injuries. Considering recent evidence from the skin, which indicates that the response of hair follicle stem cells differs depending on the magnitude of injury (Chen et al., 2015), we aimed to investigate whether this could also be true for muSCs. We have therefore investigated how Pax7-expressing cells respond to muscle injury using a transgenic zebrafish line in which the *pax7a* promoter drives eGFP expression. We have defined two protocols for creating precise muscle damage and characterized the process of injury healing using immunohistochemistry, *in situ* hybridization and *in vivo* imaging. We find that, although *pax7a*-expressing (GFP) cells in this line respond to injury, it is the extent of damage that determines whether they form new muscle fibers. Furthermore, we find that the developmental stage may have an impact on the speed of regeneration and the response of *pax7a*-expressing cells to injury.

## Materials and methods

### Zebrafish

Adult zebrafish were maintained according to standard procedures (Westerfield, 2000) in a 12 h light/dark cycle. All animal work was carried out in accordance with the Animals (Scientific Procedures) Act 1986. Embryonic fish were maintained in E3 medium at 28°C. After 5 dpf, E3 medium was changed and larvae fed with Gemma75 (Skretting) daily.

The *Tg[pax7a:eGFP]* transgenic line was a kind gift from Christiane Nüsslein-Volhardt (Max-Planck Institute for Developmental Biology, Tübingen, Germany) and has been described previously (Mahalwar et al., 2014). This line was maintained in a homozygous *pfeffer* (*pfe*, allele tm36b) mutant background (Odenthal et al., 1996). *pfe* fish form fewer *pax7a*:eGFP transgene-expressing xanthophore pigment cells in the skin due to a mutation in the *fms/csfr-1* gene (Parichy et al., 2000; Maderspacher and Nusslein-Volhard, 2003). *pax7a*:eGFP;*pfe* were crossed with double mutant *roy;mitfa* (*caspar*) mutants (White et al., 2008). The F2 offspring were maintained as homozygous *pfe;mitfa* mutants carrying the *pax7a*:eGFP transgene (subsequently referred to as *pax7a*:eGFP).

### Mechanical injury

Larvae were anesthetized in 0.01% MS-222 (Sigma) in E3 embryo medium and embedded laterally in 1.5% low melting agarose (Sigma) as previously described (Westerfield, 2000).

For small injuries, tungsten wire (diameter 0.125 mm, GoodFellow) was sharpened by electrolysis in potassium hydroxide. Wire was then cleaned and polished using a pair of fine forceps, inserted into a needle holder and fixed into a microinjection rig. Injuries were targeted to the center of the 12th ventral myotome. Approximate depth of injuries was 0.1 mm within a single myotome.

For extensive injuries, tungsten wire was replaced by an unsharpened steel manipulation needle (diameter 1 mm). Three adjacent puncture wounds were targeted to the 12th ventral myotome and the needle was agitated from a medial to distal position in order to damage as many muscle fibers as possible.

For recovery, free-swimming larvae were maintained as above. To assess proliferation, larvae were incubated in 10 mM BrdU (Sigma) 1% DMSO in E3 embryo medium for the duration of recovery.

### Immunohistochemistry

For GFP/phalloidin staining, larvae were anesthesized in 0.02% MS-222 and fixed in 4% paraformaldehyde (PFA, Sigma) for 1 h at room temperature. Samples were washed in 0.1% Tween20 in phosphate-buffered saline (PBT, Sigma) and permeabilized in 100% acetone for 30 min at −20°C. After several washes in PBT, samples were blocked in 5% goat serum (Life Technologies) in PBT for at least 2 h, then incubated with primary antibody diluted in 5% goat serum/PBT over night at 4°C. Samples were washed for at least 4 h in 0.1% PBT the following day, then incubated with secondary antibodies diluted in 5% goat serum/PBT for 2 h at room temperature. After several washes in PBT, samples were taken through glycerol series and mounted in VectaShield with DAPI (Vector Laboratories).

For Pax7/GFP staining, larvae were fixed in 2% PFA for 30 min at room temperature. Samples were washed in 1% Triton-X (Sigma) in PBS (1% PBTx) and blocked in 5% goat serum in 1% PBTx for at least 2 h. Incubation with primary antibodies, diluted in 5% goat serum/1% PBTx, was carried out for 48 h at room temperature. Samples were washed in 1% PBTx and immunostained with secondary antibody diluted in 5% goat serum/1% PBTx over night at room temperature. Larvae were then washed in 1% PBTx, taken through glycerol series and mounted in VectaShield with DAPI.

For BrdU/GFP staining, larvae were fixed for 30 min in 2% PFA at room temperature and subsequently stored in 100% methanol (Fisher) over night at −20°C. The next day, larvae were taken through a re-hydration series of methanol/PBS and washed in 1% PBTx. Following, the samples were permeabilized in 10 μg/ml of proteinase K in 0.1% PBT for 1 h at room temperature. After several washes in 0.1% PBT, samples were incubated in 2 N HCl (Sigma) in H_2_O for 1 h. Samples were subsequently washed with 1% DMSO/0.1% Tween20 in PBS (PBDT) and blocked in 5% goat serum in PBDT for at least 2 h at room temperature. Incubation with primary antibodies, diluted in 5% goat serum in PBDT, was carried out over night at 4°C. After several washes in PBDT, larvae were incubated in secondary antibodies, diluted in 5% goat serum in PBDT, for 2 h at room temperature. Samples were then washed, taken through glycerol series and mounted in VectaShield with DAPI.

Primary antibodies used: rabbit polyclonal anti-GFP (1:500; Life Technologies), mouse polyclonal anti-Pax7 (1:5, DSHB), rat monoclonal anti-BrdU (1:200, Abcam).

Secondary antibody used: goat anti-rabbit IgG AlexaFluor488 conjugated (1:500; Life Technologies), goat anti-mouse IgG AlexaFluor568 conjugates (1:500, Life Technologies), goat anti-rat IgG AlexaFluor568 conjugated (1:500, Life Technologies), fluorophore-conjugated phalloidin555 (1:300; PromoKine).

### *In situ* hybridization

*In situ* hybridization was performed as described previously (Thisse and Thisse, 2008) with the following modifications. Larvae were permeabilized in a 100 μg/ml solution of collagenase (Sigma, stock solution of 1 mg/ml in Ringer's solution, diluted 1:10 in 0.1% PBT) for 2 h at room temperature prior to hybridization with riboprobe. For hybridization, DIG-conjugated riboprobes to *myf5* (Groves et al., 2005) and *myogenin* (Weinberg et al., 1996) were used, which were detected using alkaline phosphatase conjugated FAB fragments (Roche). After detection, samples were developed in 0.25% NBT/BCIP in PBT (Sigma) for 7 days, then post-fixed in 4% PFA for 30 min, taken through glycerol series and mounted for analysis

Expression was quantified by eye and expression classified as either present or absent in the injured myotome. For all experiments, 10 larvae were used per condition and animals showing poor health after injury excluded from subsequent analyses. We then calculated the number of animals showing expression per condition as a percentage to compensate for any differences in overall number.

### Injury volume measurements

Samples were scanned using a Leica TCS SP5 microscope equipped with a Leica CTR 6500 laser and LAS AF software and subsequently analyzed using ImageJ/ Fiji (Schindelin et al., 2012). The area of injured muscle and resulting gaps between myofibers was selected using the Fiji ROI tool for each slice in a z-stack and measured using “ROI manager.” The area of each slice was then multiplied by the slice thickness and summed to obtain the total volume of injury in μm^3^.

### Individual cell tracking and counting

For time-lapsed recordings, larvae were embedded laterally in 1.5% low-melting agarose. A Nikon D-Eclipse C1 microscope with 488 nm argon laser, EZ-C1 3.70 software and x40 water dipping objective was used. Z-stacks were acquired in 1 μm steps with upper limit at the skin and lower limit at the neural tube. Z-stacks were acquired every 30 min and were 8–14 h in total duration. Time-lapsed data was processed entirely in ImageJ/Fiji. Drift was adjusted using the “Correct 3D drift” plugin, single cells were tracked manually using the “MtrackJ” plugin.

For cell counting, immunostained samples were scanned at the level of the 12th ventral myotome using a Leica confocal microscope and analyzed using Image J/Fiji. Individual DAPI+ cells in z-stacks were counted using the “Cell counter” plugin. For control larvae, cells in the uninjured 12th ventral myotome were counted.

### Data analysis

All plots and statistical analyses were generated using IBM SPSS Statistics 21.

## Results

### Small single myotome injury: a reproducible mechanical injury paradigm in zebrafish larvae

**Figure 1:**
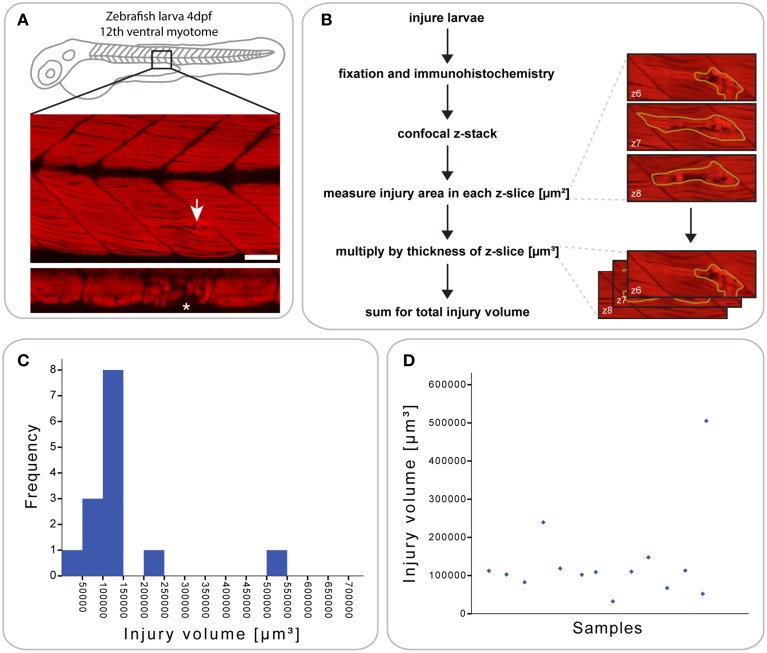
**Defining a reproducible mechanical injury paradigm in the zebrafish larva**. **(A)** Confocal slice of larvae injured in the 12th ventral myotome at 4 days post-fertilization (dpf) and immunostained for phalloidin (F-actin) at 1 h post-injury (hpi). Arrowhead indicates site of injury. Panel of orthogonal XZ view centered on the injury site is shown beneath and injury site is indicated by an asterisk. Left is anterior, top is dorsal, scale bar is 50 μm. **(B)** Workflow for the quantification of injury size. After immunostaining and confocal imaging of injured larvae, the injured muscle, and resulting gaps between myofibers were circled using Fiji ROI tool on each slice and the area measured. This is illustrated by the images shown on the right, which present the phalloidin-stained site of injury with ROI contours in yellow. The area of each slice was then multiplied by the slice thickness and all slices summed to obtain the total volume of injury in μm^3^, illustrated by the images on the right. **(C)** Histogram showing the distribution of injury volume at 1 hpi for *n* = 14 larvae injured at 4 dpf. Most samples cluster around an injury volume of 100,000 μm^3^. **(D)** Scatter plot for the same dataset, plotting the injury volume for each of the 14 samples.

Previous approaches to model muscle injury in zebrafish larvae involved the injection of cardiotoxin (chemical injury; Seger et al., 2011) and tail transection (large mechanical injury; Rodrigues et al., 2012). We were interested in investigating regeneration dynamics in response to a targeted mechanical injury to a small number of muscle fibers, and used a sharpened tungsten wire fixed into a microinjection rig. We analyzed injuries using fluorophore-conjugated phalloidin to stain F-actin in muscle fibers and so provide the means to visualize the injury. This revealed that several myofibers in the targeted myotome showed signs of tearing and hyper-contraction (indicated by brighter phalloidin staining), while all muscle fibers in neighboring myotomes were unaffected (Figure 1A). Furthermore, the injury was precisely targeted to avoid damaging the vertical or central myosepta (see asterisk in the orthogonal view in Figure 1A).

To analyze the extent and reproducibility of our small single myotome injury paradigm, we measured the total volume of the injury at 1 hours post injury (hpi) (*n* = 14 larvae; Figure 1B). As shown in (Figures 1C,D), most injury volumes were between 5 × 10^4^ and 1.5 × 10^5^ μm^3^ (11/14 larvae). Thus, a controlled injury using a sharpened tungsten wire results in a reproducible muscle wound contained within a single myotome, which can be used for evaluating the process of muscle regeneration *in vivo*. We termed this injury model a “small single myotome injury.”

### Small single myotome injuries are rapidly repaired

**Figure 2:**
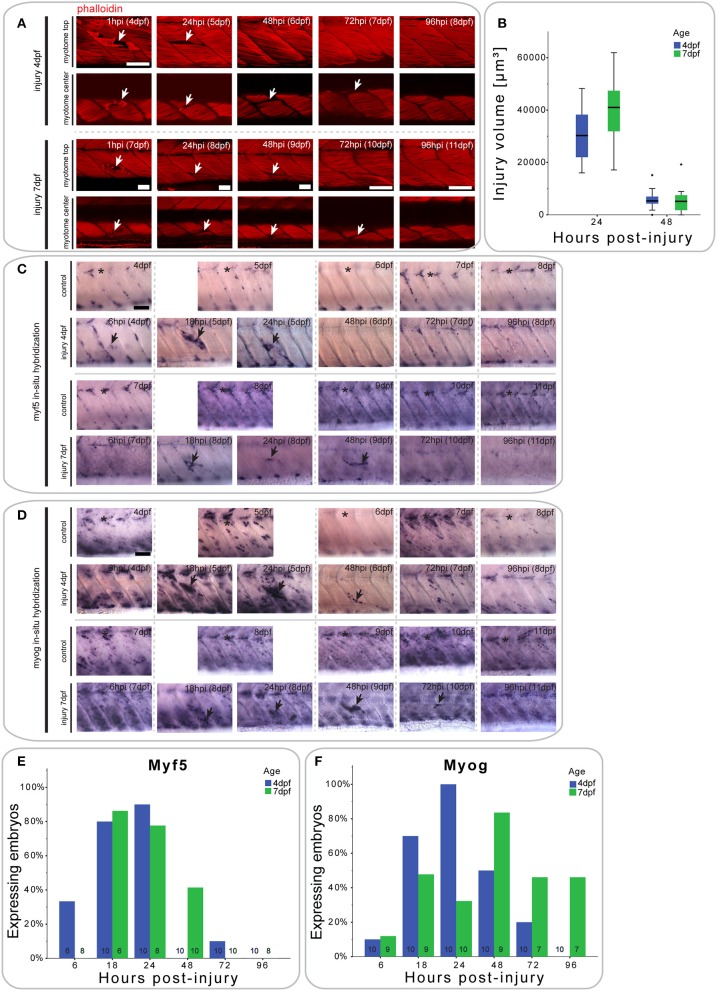
**Small single myotome injuries are rapidly repaired and result in characteristic expression of MRFs**. **(A)** Confocal slices of the superficial and deep 12th ventral myotome of larvae injured at 4 or 7 dpf and stained for phalloidin (f-actin) after injury. Arrowheads indicate site of injury. **(B)** Quantification of injury volume at varying time-points post-injury in larvae injured at 4 or 7 dpf. Injury volume was measured as described in Figure 1B. *p* < 0.005 for differences in injury volume between 24 and 48 hpi independent of age; *p* = 0.223 for differences in injury volume between 4 and 7 dpf independent of hpi; *p* = 0.158 for differences in injury volume dependent on age and hpi. **(C,D)** Representative images of *in situ* hybridization with *myf5* **(C)** or *myogenin* **(D)** anti-sense probe of uninjured controls and larvae injured in the 12th ventral myotome at 4 or 7 dpf at various time-points post-injury. The 12th myotome is marked by an asterisk in control images. Arrowheads indicate expression patterns of note in injured samples. Left is anterior, top is dorsal, scale bar is 50 μm for all images. **(E,F)** Bar graph showing the proportion of embryos expressing *myf5* **(E)** and *myogenin* **(F)** at the site of injury at different time-points post-injury. Small numbers on bars indicate the total number of larvae.

We next analyzed the size of the small single myotome injury in phalloidin-stained larvae at defined intervals after injury. Injury was performed on larvae at 4 or 7 dpf to also examine whether muscle repair was affected by developmental stage (Figure 2A). At 1 hpi, the injured myotome in 7 dpf larvae resembled that in 4 dpf fish, with hyper-contracted, and torn myofibers easily distinguishable from undamaged muscle fibers. After 24 h, large gaps were visible between myofibers in superficial and deep portions of the myotome, regardless of larva age at injury. Such gaps were never observed in adjacent, un-targeted ventral myotomes. While these gaps largely disappeared by 48 hpi in the superficial myotome of larvae injured at 4 dpf, they occasionally persisted in larvae injured at 7 dpf. Furthermore, it was noticeable that the gaps were still present in the more central regions of the myotome in larvae injured at either age. By 96 hpi, gaps in the central portion of the myotome were no longer present. Interestingly, in larvae injured at 7 dpf, the upper myotome was largely repaired by 72 hpi, but still exhibited slight distortion. To identify whether there were differences in muscle repair initiated at 4 or 7 dpf, we measured the volume of injury at 24 and 48 hpi (Figure 2B). Even though there was no significant difference between larvae injured at 4 or 7 dpf, the average injury volume of 4 dpf larvae was smaller. At 48 hpi, the injury volume of 7 dpf larvae showed a larger distribution.

### Expression profile of *myf5* during repair following small single myotome injury

Despite the visible reduction in injury size and apparent repair of the muscle, regenerating myofibers were not observed close to the original site of injury (Figure 2A). To understand how myogenesis is regulated during muscle repair, we characterized expression of MRFs using *in situ* hybridization.

Larvae injured at 4 dpf showed a small increase in *myf5* expression in the injured myotome at 6 hpi (2/6 larvae), which peaks between 18 hpi (8/10 larvae) and 24 hpi (9/10 larvae) (Figure 2C, quantified in Figure 2E). By 48 hpi, *myf5* was no longer expressed in the injured myotome (0/10 larvae).

In larvae injured at 7 dpf, *myf5* expression was not detected in the injured myotome at 6 hpi (0/10 larvae), but was elevated at 18 hpi (5/6 larvae) (Figure 2C, quantified in Figure 2E). *myf5* expression further increased at 24 hpi (6/8 larvae), though seemed less pronounced than in larvae injured at 4 dpf. Expression persisted at 48 hpi (4/10 larvae) in 7 dpf larvae, which was not the case in larvae injured at 4 dpf. After 72 hpi, *myf5* was no longer expressed in the injured myotome of 7 dpf larvae (1/10 larvae).

### Expression profile of *Myogenin* during repair following small single myotome injury

To identify cells initiating myogenic differentiation, we also investigated the expression of *myogenin* after injury. Six hours after injury, there was no noticeable *myogenin* expression in the injured myotome of 4 dpf larvae (1/10 larvae) (Figure 2D, quantified in Figure 2F). *myogenin* became detectable at 18 hpi (7/10 larvae) and was subsequently up-regulated at 24 hpi (10/10 larvae). Expression then gradually decreased between 48 hpi (5/10 larvae) and 72 hpi (3/10 larvae) and was back to background levels at 96 hpi (0/10 larvae).

Similarly, *myogenin* expression was not elevated in the targeted myotome of 7 dpf larvae at 6 hpi (1/9 larvae) (Figure 2D, quantified in Figure 2F). Interestingly, expression of *myogenin* appeared at a slower rate in the older larvae, with only slightly elevated expression at 24 hpi (3/10 larvae). Expression peaked only at 48 hpi (7/9 larvae) and persisted until 72 hpi (3/10 larvae) and 96 hpi (3/7 larvae).

Thus, repair of muscle fibers in zebrafish larvae with small single myotome injury involves an initial expression of *myf5* at the injury site, followed by *myogenin*, with *myf5* and *myogenin* persisting longer in repairing muscle in larvae injured at 7 dpf, compared to those injured at 4 dpf.

### The extensive single myotome injury model reveals that muscle regeneration dynamics are affected by injury extent

**Figure 3:**
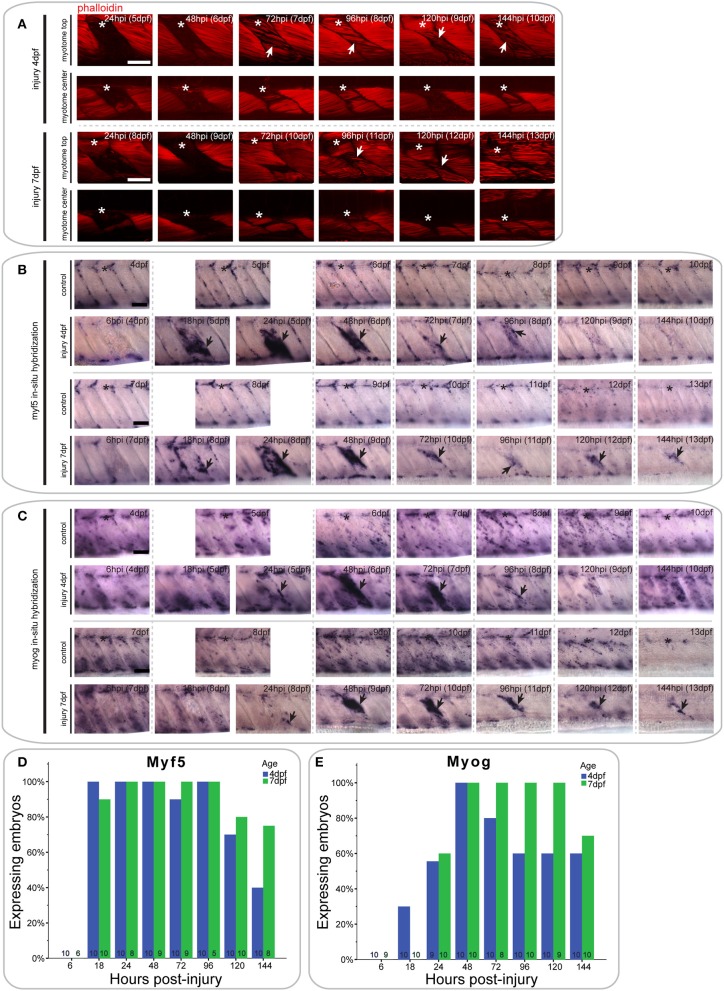
**Extensive single myotome injuries are rapidly regenerated and result in characteristic expression of MRFs**. **(A)** Confocal slices of the superficial and deep 12th ventral myotome of larvae with an extensive single myotome injury at 4 or 7 dpf and stained for phalloidin (f-actin) after injury. Asterisk indicates injured myotome, arrowheads indicate structures of note. **(B,C)**. Representative images of *in situ* hybridization using *myf5* **(B)** or *myogenin* **(C)** anti-sense probe of uninjured controls and larvae injured extensively in the 12th ventral myotome at 4 or 7 dpf at various time-points post-injury. The 12th myotome is marked by an asterisk in control images. Arrowheads indicate expression patterns of note in injured samples. Left is anterior, top is dorsal, scale bar is 50 μm for all images. **(D,E)** Bar graph showing the proportion of embryos expressing *myf5* **(D)** or *myogenin* **(E)** at the site of injury at different time-points post-injury. Small numbers on bars indicate the total number of larvae.

To test whether the size of injury affects muscle regeneration, we also developed an extensive single myotome injury paradigm, using an unsharpened steel manipulation needle which damaged at least 50% of myofibers in the targeted myotome. Muscle injuries were analyzed by staining for F-actin using fluorophore-conjugated phalloidin. Following injury at 4 dpf, no damaged muscles fibers remained in the injured myotome after 24 h (Figure 3A). The injured myotome was filled with cells as shown by DAPI labeling (data not shown). Small diameter regenerating myofibers appeared between 72 and 144 hpi. Despite regeneration of muscle fibers, the injured myotome remained mildly deformed and smaller in size than adjacent myotomes 6 days after injury.

In larvae injured at 7 dpf, most damaged myofibers were cleared by 24 hpi (Figure 3A). Regenerating myofibers first become visible between 72 and 144 hpi, though they were visibly less numerous than in larvae injured at 4 dpf. Six days post-injury, the injured myotome was still visibly distorted.

### Expression profile of *myf5* during regeneration following extensive single myotome injury

In larvae injured at 4 dpf, *myf5* expression was undetected at the site of injury at 6 hpi (0/10 larvae) (Figure 3B, quantified in Figure 3D). Expression was greatly up-regulated at 18 hpi (10/10 larvae) and remained at high levels through 24 hpi (10/10 larvae) and 48 hpi (10/10 larvae). It was also apparent in bright-field images that the size of the injured myotome was reduced at 48 hpi relative to adjacent uninjured myotomes. Expression of *myf5* decreased at 72 hpi (9/10 larvae), although it remained above endogenous levels at 96 hpi in the injured myotome (10/10 larvae). Finally, *myf5* further decreased at 120 hpi (7/10 larvae) and was reduced to levels comparable to controls by 144 hpi (4/10 larvae).

A similar profile of *myf5* expression was observed in larvae that underwent extensive single myotome injury at 7 dpf. There was no *myf5* detectable in the injured myotome at 6 hpi (0/6 larvae) (Figure 3B, quantified in Figure 3D). At 18 hpi, *myf5* expression was observed in the injured myotome, particularly around the edges (9/10 larvae). Expression increased by 24 hpi (8/8 larvae) and was robust at the site of injury at 48 hpi (9/9 larvae). As in larvae injured at 4 dpf, the size of the myotome was visibly reduced at 48 hpi. Subsequently, expression levels of *myf5* decreased but remained detectable between 72 hpi (9/9 larvae) and 96 hpi (5/5 larvae). In contrast to larvae injured at 4 dpf, larvae injured at later stages maintain higher levels of *myf5* expression at the injury site at 120 hpi (8/10 larvae) and 144 hpi (6/8 larvae).

### Expression profile of *Myogenin* during regeneration following extensive single myotome injury

The differentiation marker *myogenin* is not expressed in the injured myotome at 6 hpi in 4 dpf larvae (0/10 larvae) (Figure 3C, quantified in Figure 3E). Slight expression was visible at 18 hpi (3/10 larvae) and 24 hpi (5/9 larvae). *myogenin* expression was then greatly increased at 48 hpi (10/10 larvae), and remained at a high level through to 72 hpi (8/10 larvae). *Myogenin* expression was reduced at 96 hpi (6/10 larvae) and then remained at a constant level in the regenerating myotome through 120 hpi (6/10 larvae) and 144 hpi (6/10 larvae).

Larvae injured at 7 dpf had a very similar *myogenin* expression profile to those injured at 4 dpf. There was little detectable expression in the injured myotome at 6 hpi (0/9 larvae) and 18 hpi (0/10 larvae) (Figure 3C, quantified in Figure 3E). Small groups of cells started to express *myogenin* around the site of injury at 24 hpi (6/10 larvae). Expression then strongly increased between 48 hpi (10/10 larvae) and 72 hpi (8/8 larvae). Compared to larvae injured at 4 dpf, *myogenin* expression was stronger at 96 hpi and still prominent around the wound site (10/10 larvae). Similarly, expression levels of *myogenin* were still markedly elevated at 120 hpi (9/9 larvae) and 144 hpi (7/10 larvae) in larvae injured at 7 dpf.

Thus, larvae injured at 4 or 7 dpf are both able to partially regenerate muscle following an extensive single myotome injury, which damages the majority of a myotome. However, larvae injured at 7 dpf appear to regenerate fewer myofibers than those injured at 4 dpf and show prolonged expression of MRFs throughout the course of regeneration.

### *pax7a*-expressing cells in the myotome display developmental-dependent changes in their behavior

We hypothesized that the delayed regenerative response in 7 dpf larvae relative to that in 4 dpf larvae could be caused by a developmental change in muSC behavior. To investigate this, we used a transgenic line in which eGFP is expressed under control of a *pax7a* promoter (*pax7a*:eGFP; Mahalwar et al., 2014). In this line, we note that there were many GFP+ cells localized at the myoseptum, where Pax7+ cells have previously been described during zebrafish development and have been shown to be recruited to sites of muscle injury (Devoto et al., 2006; Hollway et al., 2007; Seger et al., 2011).

**Figure 4:**
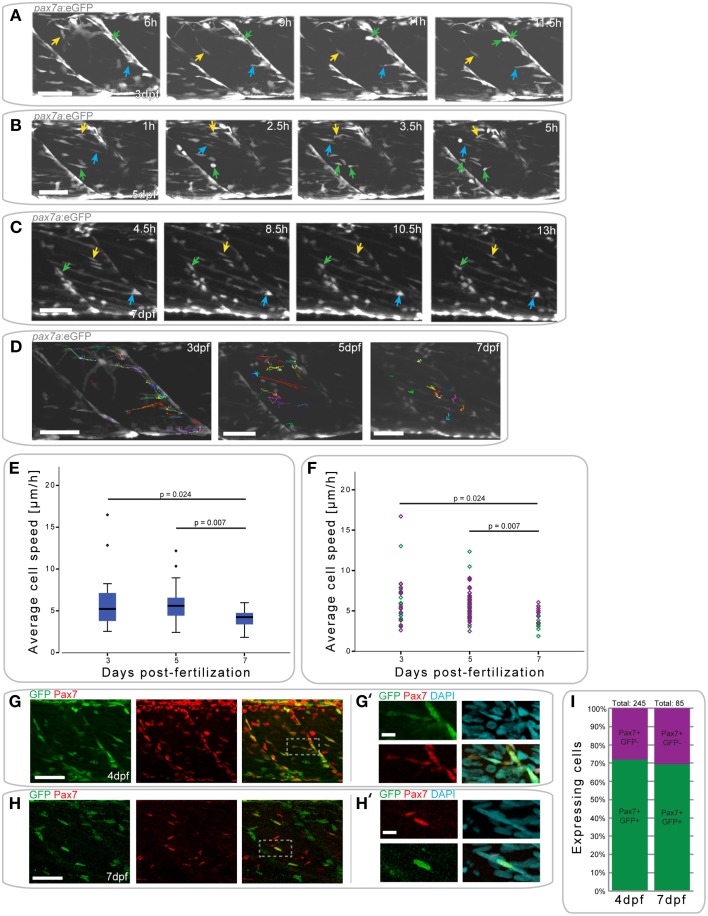
*****pax7a***+ cells in the myotome display changes in their behavior depending on developmental stage**. **(A)** Z-projections of selected time-points captured every 30 min after the start of a confocal time-lapse stack of an uninjured *pax7a*:eGFP larva at 3 dpf. Arrowheads of different colors indicate cells which show characteristic cell movement throughout the time-lapse. **(B,C)** Z-projections of selected time-points after the start of a confocal time-lapse stack of an uninjured *pax7a*:eGFP larva at 5 dpf **(B)** and 7 dpf **(C)**. Arrowheads of different colors indicate cells which show characteristic cell movement throughout the time-lapse. **(D)** Z-projections of the last frame of time-lapse recordings shown in **(A–C)**. Individual cells were manually tracked throughout the duration of time-lapse recordings using Fiji MtrackJ. Colored and numbered lines indicate the movement of individual cells through the entire duration of the time-lapse for 3, 5, and 7 dpf larvae. **(E)** Box plot indicating the average velocity of cells tracked in *n* = 2 time-lapse recordings in 3, 5, and 7 dpf larvae. *P*-values were calculated using One-Way ANOVA with Tukey *post-hoc* test. **(F)** Scatter plot indicating the average velocity of individual cells in *n* = 2 timelapse recordings in 3, 5, and 7 dpf larvae. Each diamond represents one cell; different colors indicate the n-number, thus different individual larvae. *P*-values were calculated using One-Way ANOVA with Tukey *post-hoc* test. **(G,H)** Z-projections of 4 dpf **(G)** or 7 dpf **(H)** *pax7a*:eGFP larvae immunostained for GFP (green) and Pax7 (red). **(G**′**,H**′**)** Magnified images of boxed region in **(G)** or **(H)**, respectively with addition of DAPI image (cyan) and GFP/Pax7/DAPI merge. Left is anterior, top is dorsal, scale bar is 50 μm for images of myotome, 10 μm for magnified images. **(I)** Percentage bar chart indicating the proportion of *pax7a*-expressing cells which are GFP+ in un-injured 4 (*n* = 12) and 7 dpf (*n* = 12) larvae.

To first understand how *pax7a*+ cells behave during different stages of muscle development, we characterized GFP+ cell movement in *pax7a*:eGFP larvae between 3 and 7 dpf using time-lapsed microscopy (Figures 4A–C, Videos S1–S3).

At 3 dpf, GFP+ cells in *pax7a*:eGFP larvae were located at the horizontal and vertical myosepta as previously described for Pax7+ cells (Hollway et al., 2007; Seger et al., 2011). GFP+ cells at the vertical myosepta could occasionally be observed moving into the middle of the myotome, although the majority of the cells remained at the myosepta (Figure 4A). This movement was preceded by the extension of processes toward the middle of the myotome (Figure 4A, blue arrow), followed by cell migration (Figure 4A, yellow arrow). Cell division events could be observed at this stage (mean = 0.375 divisions/h, stdev = 0.177 for *n* = 2 larvae) and predominantly occurred at the myosepta (Figure 4A, green arrow).

In 5 dpf *pax7a*:eGFP larvae, there was a more extensive movement of GFP+ cells (Figure 4B). Fewer cells appeared to be present at the myosepta and cells could be observed moving from this area toward the middle of the myotome (Figure 4B, yellow arrow). In the middle of the myotome, cells appeared to be moving along, or adjacent to, muscle fibers (Figure 4B, blue arrow). Further, cell division events occurred at 5 dpf (mean = 0.5 divisions/h, stddev = 0.303 for *n* = 2 larvae), but in the middle of the myotome (Figure 4B, green arrow; also in the upper left portion of the myotome at 5 h).

GFP+ cell behavior differs entirely in *pax7a*:eGFP larvae at 7 dpf (Figure 4C). Cells were located both at the myosepta and in the middle of the myotome, but did not show extensive movement, unlike at 3 or 5 dpf. Cells at the myosepta were generally of a rounded morphology and occasionally extended processes toward the middle of the myotome, but did not move from their myoseptal location (Figure 4C, green and blue arrows). On the contrary, cells located in the middle of the myotome had an elongated morphology, aligned in the same orientation as muscle fibers. These cells did not move extensively along the myofibers, but occasionally their cell morphology changed (Figure 4C, yellow arrow). Furthermore, in contrast to 3 and 5 dpf, no cell divisions could be observed in 7 dpf larvae (mean = 0 for *n* = 2 larvae).

To quantify GFP+ cell behavior during development in *pax7a*:eGFP larvae, cell movement in the myotome was analyzed in 3, 5, and 7 dpf larvae through manual tracking (Figures 4D–F). This revealed that the average cell speed did not differ significantly between 3 and 5 dpf larvae (*p* = 0.959), but was significantly reduced in 7 dpf larvae (3 dpf vs. 7 dpf: *p* = 0.024; 5 dpf vs. 7 dpf: *p* = 0.007). Furthermore, there was less variability in the average cell speed of GFP+ cells at 7 dpf relative to 3 or 5 dpf.

In summary, *pax7a*+ cells display different cell behaviors at 3, 5, and 7 dpf, with changes in cell migratory behavior and location.

To confirm that GFP expression in cells of the myotome of *pax7a*:eGFP larvae occurs in Pax7-expressing cells, we performed immunohistochemistry on 4 and 7 dpf larvae to detect GFP and Pax7 (Figures 4G–H′) and counted the number of labeled cells in the myotome (Figure 4I). This analysis shows that approximately 70% of Pax7+ cells also express the GFP protein in both 4 and 7 dpf larvae (Figure 4I). Further, we note that approximately 92% of GFP+ cells also express Pax7 at 4 dpf, with the remaining 8% accounting for freshly divided or differentiating cells (data not shown). We are thus confident that the *pax7a*:eGFP line is an accurate reporter of Pax7 protein expression in the myotome.

### *pax7a*+ cells show differential responses to muscle injury dependent on age

Pax7+ SCs are the principal cell type that regenerates muscle in mammals (Relaix and Zammit, 2012), but it is not clear if this is true for zebrafish. To characterize the response of *pax7a*+ cells to muscle injury in zebrafish, we generated a small single myotome injury in 4 and 7 dpf *pax7a*:eGFP larvae and observed GFP+ cell responses by time-lapsed recordings from 30 min after injury (Videos S4, S5).

**Figure 5:**
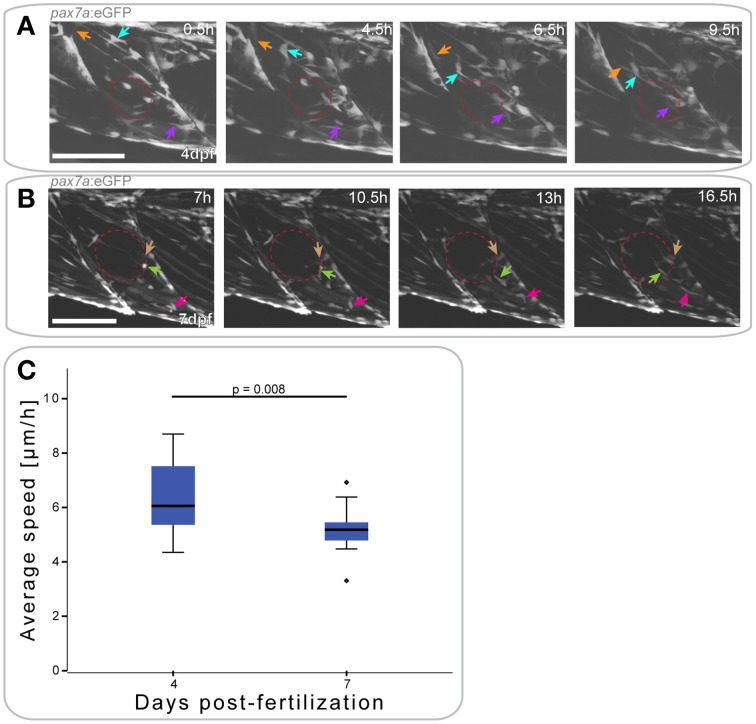
*****pax7a***+ cells show differential responses to muscle injury dependent on age**. **(A,B)** Z-projections of selected time-points after the start of a confocal time-lapse stack of a *pax7a*:eGFP larva injured at 4 dpf **(A)** or 7 dpf **(B)**. Recording was started 30 min after injury and stacks captured every 30 min. Red ellipse indicates the site of injury, arrowheads of different colors indicate cells which show characteristic cell movement throughout the time-lapse. Left is anterior, top is dorsal, scale bar is 100 μm for all images. **(C)** Box plot indicating the average velocity of cells tracked in time-lapse recordings of larvae injured at 4 or 7 dpf (*n* = 2 at each stage). *P*-values were calculated using Student's *t*-Test.

In *pax7a*:eGFP larvae injured at 4 dpf, the first GFP+ cell responses (extension of processes) were observed as soon as 4 hpi (Figure 5A); cells moved toward the injury from both vertical and horizontal myosepta (Figure 5A, orange and purple arrows). At the site of injury, GFP+ cells then elongated in the same orientation as existing fibers between 9 and 14 hpi. GFP+ cells located in the middle of the myotome responded to the injury in the same manner as those at the myosepta, displaying a migratory response to the injury (Figure 5A, turquoise arrow).

Muscle injury in 7 dpf *pax7a*:eGFP larvae resulted in GFP+ cells extending thin processes toward the injury around 7 hpi (Figure 5B, maroon arrow). Cell movement toward the site of injury was not visible until approximately 10 hpi. At this stage, cells adjacent to the injury site (Figure 5B, light green arrow) and at more distal locations (Figure 5B, pink arrow) adopted an elongated morphology.

To determine whether cell responses to injury are affected by developmental stage, we again tracked GFP+ cells. Average cell speed of only those cells that responded to the injury was measured and plotted (Figure 5C). This revealed that GFP+ cells responding to muscle injury in 7 dpf larvae were significantly slower than those in 4 dpf larvae (*p* = 0.008).

### *pax7a*-expressing cells do not contribute to fiber formation after small single myotome injury

The response of GFP+ cells to muscle injury in *pax7a*:eGFP larvae suggests that they may contribute to muscle repair. To test this, we investigated whether GFP+ cells can contribute to myofiber formation after injury, by assessing whether GFP was localized to F-actin+ muscle fibers labeled with fluorophore-conjugated phalloidin.

**Figure 6:**
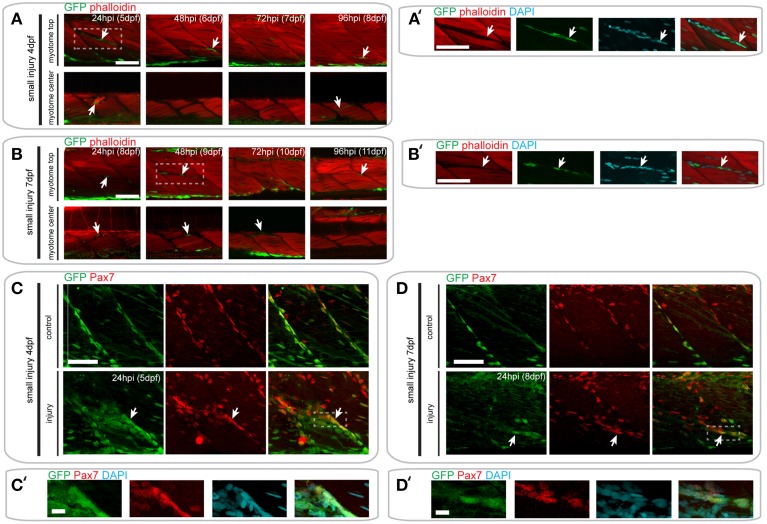
*****Pax7a***-expressing cells do not contribute to muscle fiber formation after small single myotome injury**. **(A,B)** Confocal slices of the superficial and deep 12th ventral myotome of *pax7a*:eGFP larvae injured at 4 dpf **(A)** or 7 dpf **(B)** and stained for phalloidin (f-actin, red) and GFP (green) after small single myotome injury. Arrowheads indicate cells of interest. **(A**′**,B**′**)** Magnified images of boxed regions in **(A)** or **(B)**, respectively. Images show staining for phalloidin (f-actin, red), GFP (green) and DAPI (cyan). Arrowheads indicate cells of interest. **(C,D)** Z-projections of 4 dpf **(C)** or 7 dpf **(D)** *pax7a*:eGFP larva immunostained for GFP (green) and Pax7 (red) at 24 hpi after small single myotome injury. **(C**′**,D**′**)** Magnified images of boxed region in **(C)** or **(D)**, respectively with addition of DAPI image (cyan) and GFP/Pax7/DAPI merge. Scale bar is 10 μm. For all other images, scale bars are 50 μm. Left is anterior, top is dorsal.

In *pax7a*:eGFP larvae with a small single myotome injury at 4 dpf, GFP+ cells were clearly localized to the site of injury by 24 hpi (Figures 6A,A′). Interestingly, these cells were clustered in the gaps between myofibers observed after injury, although many GFP- cells were also present (data not shown). However, no GFP+/F-actin+ striated muscle fibers were detected at the injury site, which would be indicative of myofibers derived from cells expressing the *pax7a:eGFP* transgene. Despite this absence of GFP+ F-actin+ fibers, GFP+ cells could be seen in close proximity to each other and sometimes with multiple DAPI labeled nuclei at 24 hpi (7/9 samples), which may indicate fusion (Figure S1A). GFP+ cells were no longer present in clusters by 48 hpi, coincident with loss of the spaces between myofibers. Again, no GFP+/F-actin+ muscle fibers were detected at this stage. By 72 hpi, GFP+ cells were found between myofibers, similar to those in uninjured myotomes.

In *pax7a:eGFP* larvae injured at 7 dpf, fewer GFP+ cells were observed at the wound site at 24 hpi, relative to those injured at 4 dpf. Clusters of GFP+ cells could be observed at the wound site at both 24 and 48 hpi when larvae were injured at 7 dpf (Figures 6B,B′). Similarly to 4 dpf larvae, GFP+ cells lay close to each other and could sometimes be identified as multinuclear (4/5 samples; Figure S1B). At 72 hpi, these GFP+ cells were still present in the deep portions of the myotome, but disappeared in the superficial layers. We were not able to observe GFP+/F-actin+ fibers at any of these time-points in 7 dpf larvae, similar to our findings from 4 dpf larvae.

We confirmed these findings using GFP/Pax7 co-immunostaining at 24 hpi in larvae injured at 4 or 7 dpf (Figures 6C–D′). This shows that many cells that are localized to the site of injury also express the Pax7 protein.

### *pax7a*-expressing cells contribute to fiber formation after extensive single myotome injury

Since we were unable to identify GFP+ muscle fibers in small single myotome injured *pax7a*:eGFP larvae, we wondered whether the size of injury could influence the ability of *pax7a*+ cells to contribute to muscle regeneration. We tested this by inducing an extensive single myotome injury in 4 and 7 dpf larvae and evaluated the ability of GFP+ cells to form myofibers at different times after injury.

**Figure 7:**
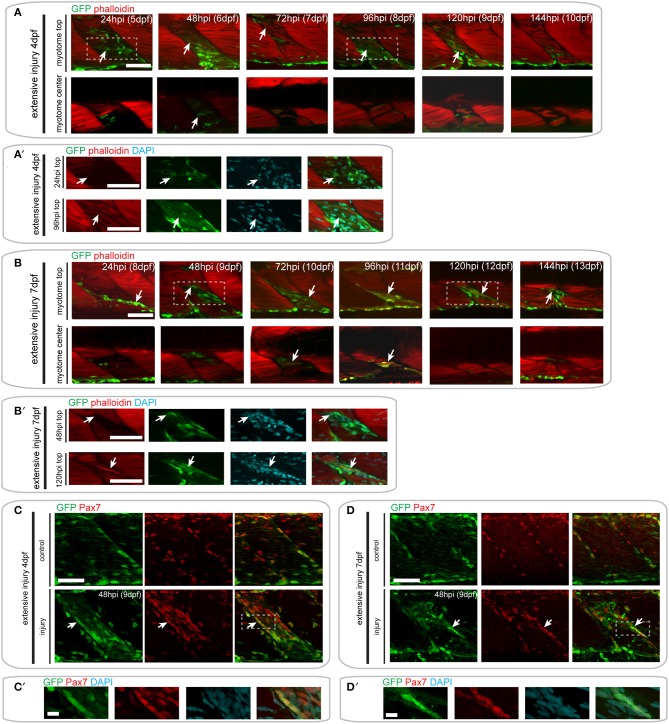
*****Pax7a***-expressing cells contribute to muscle fiber formation after extensive single myotome injury**. **(A,B)** Confocal slices of the superficial and deep 12th ventral myotome of *pax7a*:eGFP larvae injured extensively at 4 dpf **(A)** or 7 dpf **(B)** and stained for phalloidin (f-actin, red) and GFP (green) after injury. Arrowheads indicate cells of interest and regenerating GFP-positive fibers. **(A**′**,B**′**)** Magnified images of boxed regions in **(A)** or **(B)**, respectively. Images show staining for phalloidin (f-actin, red), GFP (green) and DAPI (cyan). Arrowheads indicate cells of interest. **(C,D)** Z-projections of 4 dpf **(C)** or 7 dpf **(D)** *pax7a*:eGFP larvae immunostained for GFP (green) and Pax7 (red) at 48 hpi after extensive single myotome injury. **(C**′**,D**′**)** Magnified images of boxed region in **(C)** or **(D)**, respectively with addition of DAPI image (cyan) and GFP/Pax7/DAPI merge. Scale bars are 10 μm. For all other images, scale bars are 50 μm. Left is anterior, top is dorsal.

Following injury of 4 dpf *pax7a:eGFP* larvae, we observed numerous GFP+ cells around the site of injury at 24 hpi (Figures 7A,A′). Elongated GFP+ cells were first observed at 48 hpi, and GFP+/F-actin+ fibers were first observed at 72 hpi (Figure 7A′). Interestingly, we noted that some of the regenerating fibers in the injured myotome were not GFP+ (Figure 7A, see 72 hpi arrow). GFP+ cells were still present within the injured myotome at 144 hpi, but were less numerous than at earlier stages of injury.

A similar response of GFP+ cells was observed in *pax7a:eGFP* larvae injured extensively at 7 dpf (Figure 7B). GFP+ cells were present in the injured myotome at 24 and 48 hpi and seemed to lie in the same orientation as existing muscle fibers (Figure 7B′). The first GFP+/F-actin+ fibers appeared by 72 hpi and were detectable until 120 hpi in the injured myotome. By 144 hpi, GFP+ cells were still present within the myotome, though GFP+ fibers could no longer be observed (Figure 7B).

Again, we confirmed the presence of Pax7-expressing cells at the site of injury at 48 hpi using GFP/Pax7 immunohistochemistry (Figures 7C-D′). This staining shows that there is a large number of Pax7-expressing cells present at the site of injury in larvae injured at 4 and 7 dpf. Many of these cells also expressed GFP, indicating that the *pax7a*+ cells contributing to regeneration are expressing Pax7 protein.

### A pool of *pax7a*-expressing cells is recruited for repair and regeneration in 7 dpf, but not 4 dpf animals

To assess whether *pax7a*-expressing cells proliferated in response to injury, similar to mammals, we pulsed *pax7a:eGFP* larvae after injury with BrdU and counted GFP+ and BrdU+ cells after immunostaining.

**Figure 8:**
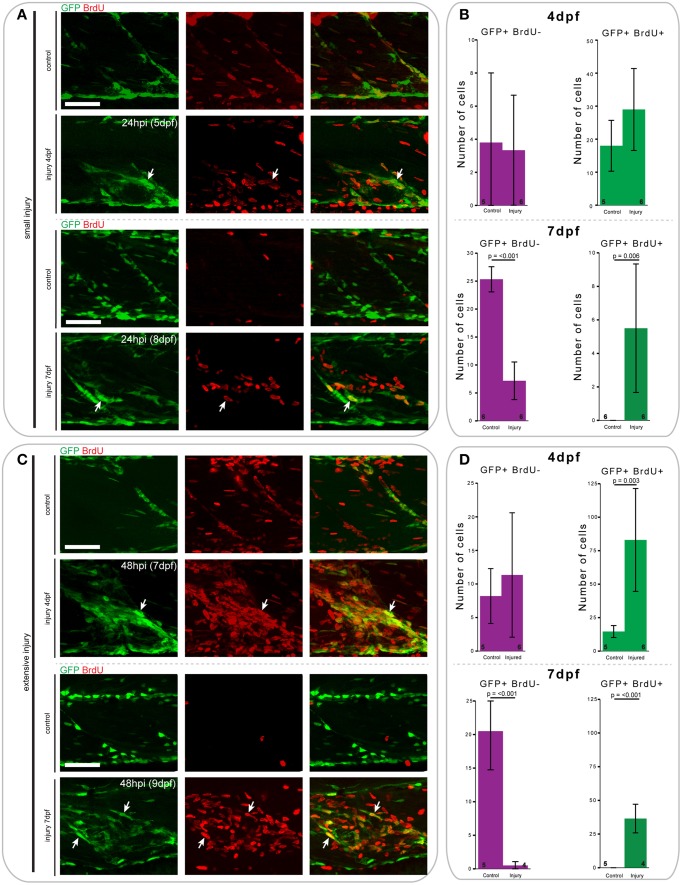
**Proliferation patterns of ***pax7a***-expressing cells differ between 4 and 7 dpf larvae following small and extensive single myotome injury**. **(A,C)** Z-projections of 4 and 7 dpf *pax7a*:eGFP larva immunostained for GFP (green) and BrdU (red) at 24 hpi after small **(A)** or extensive **(C)** single myotome injury. Larvae were incubated in 10 mM BrdU/1% DMSO during the entirety of the recovery period. Arrows indicate cells which show double staining. Scale bars are 50 μm. Left is anterior, top is dorsal. **(B,D)** Bar charts indicating the average number of GFP+BrdU- or GFP+BrdU+ cells after small **(B)** or extensive **(D)** single myotome injury compared to un-injured controls. Single cells were counted at 24 hpi **(B)** or 48 hpi **(D)** in larvae injured at 4 or 7 dpf. Numbers of larvae counted are indicated in small font at the bottom of each bar.

At 24 h after creation of a small single myotome injury in 4 and 7 dpf *pax7a:eGFP* larvae, there was clearly observable cell proliferation (Figures 8A,B). Upon injury, the number of GFP+BrdU+ cells increased 0.7-fold in 4 dpf larvae, whereas the number of GFP+BrdU− cells did not obviously change. In 7 dpf larvae we observed a significant increase in the number of proliferating GFP+ cells after injury (*p* = 0.006), which was coincident with a sharp decrease in the number of BrdU− cells (*p* < 0.001).

Similar trends can be observed 48 h after extensive single myotome injury (Figures 8C,D). In *pax7a:eGFP* larvae injured at 4 dpf, there was a significant increase in the number of proliferating GFP+ cells compared to controls (*p* = 0.003). We also observed a slight increase in the number of GFP+BrdU- cells at this stage. In 7 dpf larvae, the increase in the number of proliferating GFP+ cells was even more pronounced than for small single myotome injuries (*p* < 0.001). Further, we observed the same reduction in number of GFP+BrdU− cells as seen occurring after small injuries (*p* < 0.001).

When comparing the average number of counted cells in 4 and 7 dpf *pax7a:eGFP* larvae, it is notable that there were consistently more GFP+BrdU+ cells present in 4 dpf larvae compared to 7 dpf. Of further interest, we found that most GFP+ cells were proliferative at 4 dpf in uninjured larvae, whereas no GFP+ cells showed BrdU incorporation in uninjured 7 dpf larvae.

Thus, we observe significant differences in the proliferation patterns of GFP+ cells in response to small and extensive single myotome injury between 4 and 7 dpf *pax7a:eGFP* larvae.

## Discussion

Skeletal muscle regeneration is a complex process, involving the activation and differentiation of stem cells and their integration into existing muscle tissue or formation of *de novo* myofibers. In this study, we characterize the response of a population of muscle-resident *pax7a*+ cells during muscle injury and repair in zebrafish. We describe two reproducible mechanical injury models, resulting in minor or extensive damage to muscle fibers. We show these injuries are repaired and involve the expression of MRF genes, as seen in other vertebrates. Such injuries elicit the response of a population of *pax7a*-expressing cells resident in the myotome, which migrate to sites of muscle injury in a transgenic *pax7a:*eGFP line. The contribution of *pax7a*-expressing (GFP+) cells to regenerating muscle was dependent on the injury extent: they contributed to fiber formation after extensive single myotome injury, whereas a small injury did not lead to participation of *pax7a*-expressing (GFP+) cells in fiber repair. We also found that developmental stage was important for the speed of these cells and the duration of MRF expression in the regenerative phase. Overall, this highlights the diverse response of a muscle-resident *pax7a*-expressing cell population to injury and suggests that their role during regeneration can be affected by several parameters.

### Variations in injury size might alter muSC behavior during regeneration

Little is known about the behavior of muscle progenitor cells during the regeneration of skeletal muscle in zebrafish. In larval muscle, it has been shown that Pax3/Pax7-expressing cells are proliferative and accumulate around the site of injury (Seger et al., 2011). We observe that *pax7a*+ cells migrate toward the wound site through the extension of cytoplasmic processes, though we do not see evidence of proliferation using time-lapsed imaging. This mode of migration may in part be regulated by Rho/Rac GTPases (Murali and Rajalingam, 2014). On the contrary, in an *in vitro* single fiber system, mammalian SCs have been described to migrate through blebbing (Otto et al., 2011). This process also appears to be dependent on Rho kinase (ROCK) function, as application of small molecule inhibitors caused cells to display a slower movement (Collins-Hooper et al., 2012). Although we cannot clearly discriminate between a mesenchymal or amoeboid cell movement, we note that migrating *pax7a*-expressing cells *in vivo* clearly extend cytoplasmic processes.

Initially, we hypothesized that *pax7a*-expressing cells in injured larvae would contribute to tissue regeneration, as seen in mammals. However, we were unable to verify any contribution of GFP+ cells to fiber formation after small injury in *pax7a*:eGFP larvae. An earlier study, in which a different *pax7a*:GFP line was used, likewise did not show a contribution of GFP+ cells to regenerating fibers following cardiotoxin injection (Seger et al., 2011). Despite this, MRFs are expressed in response to small single myotome injury and GFP+ cells proliferate and migrate to the site of injury. Further, the presence of multi-nuclear and closely associated GFP+ cells is strongly indicative that fusion occurs in our small injury model. Perhaps, the number of *pax7a*-expressing cells contributing to repair in this injury model is not extensive, preventing detection of residual GFP protein in newly regenerated fibers. Alternatively, other cell populations may play a more prominent role in the context of small injuries. For instance, zebrafish also possess a paralog of the *pax7a* gene, named *pax7b*. The Pax7 antibody used in this study is unable to distinguish between these two Pax7 isoforms. Examination of fish models with a reporter gene targeted to the *pax7b* locus would be useful to determine the contribution of this isoform to muscle repair in the context of small injuries.

In mouse, Pax7+ cells are crucial for muscle to sustain its regenerative ability (Relaix and Zammit, 2012). Many muscle-resident cell populations, which do not express Pax7, have previously been shown to be able to contribute to muscle regeneration, such as side-population cells (Gussoni et al., 1999; Asakura et al., 2002), muscle-derived stem cells (Qu-Petersen et al., 2002) and CD133+ progenitor cells (Torrente et al., 2004). Even though these particular cell populations have not been described in zebrafish, it is possible that such a non-*pax7*-expressing cell population acts to repair small injuries to the muscle tissue, whereas *pax7a*-expressing cells only regenerate muscle following larger insult.

*In vitro* data from a related species of cyprinid, *Devario aequipinnatus* or giant Danio, shows that Pax7 is actually only expressed in newly activated muSCs, whereas Pax3 is present throughout myoblast proliferation (Froehlich et al., 2013). It is thus possible that *pax3*-expressing cells or other myogenic progenitor cells in fish might actually resemble a mammalian muSC population more closely than the cells we observed in our work. Considering our rudimentary understanding of sub-populations of muSCs and their roles in regeneration, especially in humans (Boldrin et al., 2010), studying these in zebrafish might lead to new insights other animal models cannot provide. We have not examined the role of *pax3*-expressing cells during muscle regeneration in zebrafish, though it is well-established that Pax3 acts to specify early muscle progenitor cells in mouse (Schubert et al., 2001). Given that Pax3 and Pax7 are related transcription factors that are both important for specifying muscle progenitor cell populations, it is possible that Pax3 may also be important for regeneration in zebrafish.

Proteins of the muscle extra-cellular matrix, such as the dystrophin-associated protein complex and laminins, are localized at the myosepta in zebrafish (Gupta et al., 2012; Wood and Currie, 2014). In accordance with prior findings (Seger et al., 2011), we note that the *pax7a*-expressing cells in the myotome of larval fish are localized to the myosepta, suggesting it is an environment that maintains this cell population. Given that we observe many *pax7a*-expressing cells with low levels of division by 7 dpf, it is tempting to speculate the myoseptum may act as a niche for these cells. Our extensive single myotome injury paradigm may indeed perturb the myoseptum, which could explain the differential regeneration response we see with respect to injury size. In this context, it is conceivable that signals from the myoseptum dictate the response of *pax7a*-expressing cells to injury.

### Variations in age might alter muSC behavior during regeneration

The difference in the dynamics of regeneration between larvae injured at 4 and 7 dpf is very consistent. Most interestingly, GFP+ cells in *pax7a*:eGFP larvae migrate toward the injury significantly slower when injured at 7 dpf compared to 4 dpf. *In vitro* studies of SCs isolated from mouse have previously shown that older cells show slower migration along myofibers (Collins-Hooper et al., 2012). The amount of GFP+ cells that proliferate in response to injury is also consistently lower in 7 dpf larvae compared to 4 dpf larvae. Further, we observe prolonged expression of MRFs and fewer regenerative fibers in larvae injured at 7 dpf relative to 4 dpf. These observations suggest that the cell populations contributing to muscle regeneration in older animals are less efficient or slower in their responses. It is possible that *pax7a*-expressing cells at 4 dpf may be more developmentally plastic or poised, thus more prone to respond to stimuli than at later stages. This phenomenon might well-reflect the entry of *pax7a*-expressing cells into quiescence after the completion of developmental myogenesis. This is also supported by our observation that we did not observe GFP+ cells undergoing proliferation in uninjured 7 dpf *pax7a:eGFP* animals, whereas there is ample proliferation in uninjured 4 dpf control fish.

It has been suggested that stem cell quiescence may be caused by the accumulation of histone marks, which promote the formation of heterochromatin, thus inhibiting gene expression (Grigoryev et al., 2004; Srivastava et al., 2010). Furthermore, the chromatin of muSCs shows an increased deposition of inhibitory histone marks during aging, which may correlate with impaired muSC function in regeneration (Liu et al., 2013). It is possible that similar mechanisms of epigenetic gene inactivation lead to decreased muSC activity between 4 and 7 dpf, explaining the difference in cell speed and regenerative responses.

Alternatively, the changes in cell behavior we observed may reflect an alteration of the environment of muSCs, such as changes to the ECM. Indeed, significant changes occur to the ECM between 3 and 6 dpf larvae (Charvet et al., 2011). Most notably, collagen organization changes from a loose, disorganized meshwork into a dense, regular network of collagen fibrils. Since it is well-known that the cellular environment of SCs can greatly impact their behavior (Montarras et al., 2013), it is conceivable that this maturation of the ECM in the myotome may lead to changes in *pax7a*-expressing cell response to injury and thus affects the speed of tissue regeneration.

## Conclusion

Our finding that responses of *pax7a*-expressing cells are dictated by the extent of the muscle injury are intriguing, especially since most injury models in mouse, such as cardiotoxin injection or crush injuries, result in a severe disruption of the tissue, leading to extensive regeneration. The impact of age on the behavior of muSCs in zebrafish and their efficiency in regenerating muscle also provides a new consideration for the study of muSC plasticity.

### Conflict of interest statement

The authors declare that the research was conducted in the absence of any commercial or financial relationships that could be construed as a potential conflict of interest.

## Supplementary material

Video S1 **Z-projection of a confocal time-lapse stack of an uninjured ***pax7a***:eGFP larva at 3 dpf**. Stacks were captured every 30 min in 1 μm slices from skin to neural tube. Left is anterior, top is dorsal.

Video S2 **Z-projection of a confocal time-lapse stack of an uninjured ***pax7a***:eGFP larva at 5 dpf**. Stacks were captured every 30 min in 1 μm slices from skin to neural tube. Left is anterior, top is dorsal.

Video S3 **Z-projection of a confocal time-lapse stack of an uninjured ***pax7a***:eGFP larva at 7 dpf**. Stacks were captured every 30 min in 1 μm slices from skin to neural tube. Left is anterior, top is dorsal.

Video S4 **Z-projection of a confocal time-lapse stack of ***pax7a***:eGFP larva injured at 4 dpf**. Capture was started 30 min after injury and stacks were captured every 30 min in 1 μm slices from skin to neural tube. Left is anterior, top is dorsal.

Video S5 **Z-projection of a confocal time-lapse stack of ***pax7a***:eGFP larva injured at 7 dpf**. Capture was started 30 min after injury and stacks were captured every 30 min in 1 μm slices from skin to neural tube. Left is anterior, top is dorsal.

Figure S1 **(A,B) Confocal slices of the 12th ventral myotome of ***pax7a***:eGFP larvae injured at 4 dpf (A) or 7 dpf (B) and stained for phalloidin (f-actin, red), GFP (green), and DAPI (cyan) 24 h after small injury**. Arrowheads indicate cells of interest, which appear to be multinuclear. Scale bars are 25 μm. Left is anterior, top is dorsal.
