# Supplementary figures and images for: A population of Pax7-expressing muscle progenitor cells show differential responses to muscle injury dependent on developmental stage and injury extent
Source: Front Aging Neurosci. 2015 Aug 25;7:161. doi: 10.3389/fnagi.2015.00161 (PMC4548158; doi:10.3389/fnagi.2015.00161)

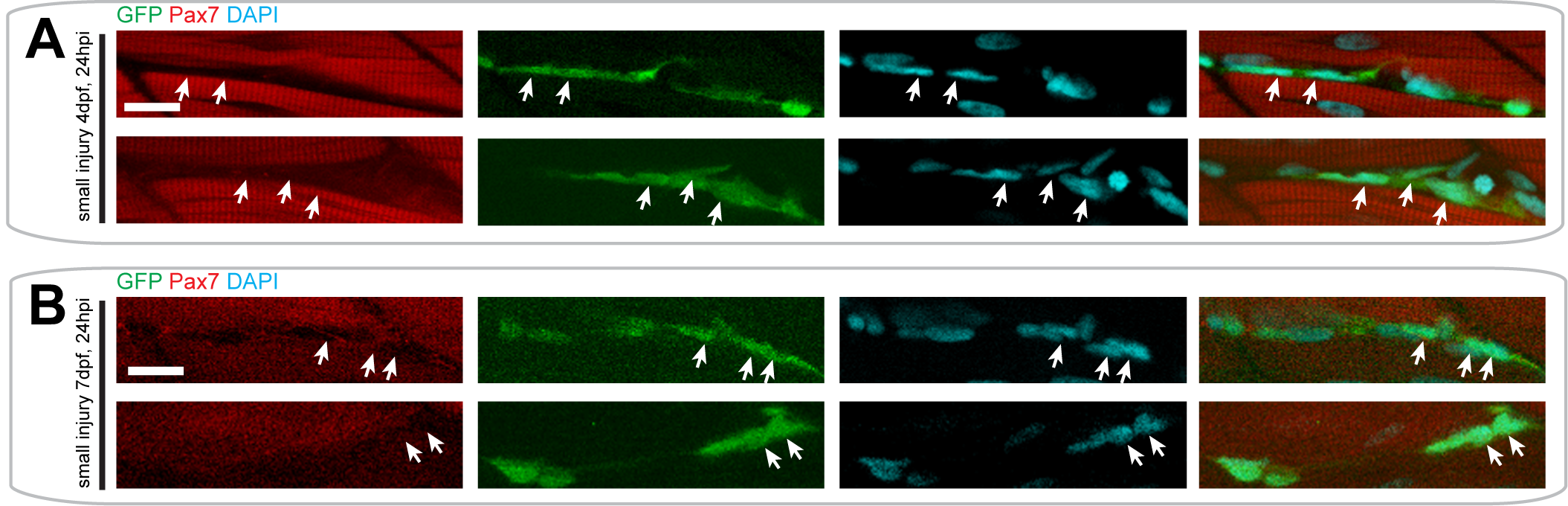

Supplement: Figure S1 — (A,B) Confocal slices of the 12th ventral myotome of pax7a:eGFP larvae injured at 4 dpf (A) or 7 dpf (B) and stained for phalloidin (f-actin, red), GFP (green), and DAPI (cyan) 24 h after small injury. Arrowheads indicate cells of interest, which appear to be multinuclear. Scale bars are 25 μm. Left is anterior, top is dorsal. [file Image1.TIF]
